# Longitudinal Physical Activity Change During Hemodialysis and Its Association With Body Composition and Plasma BAIBA Levels

**DOI:** 10.3389/fphys.2019.00805

**Published:** 2019-06-25

**Authors:** Alessio Molfino, Maria Ida Amabile, Thomas Ammann, Silvia Lai, Alessandra Grosso, Luana Lionetto, Alessandra Spagnoli, Maurizio Simmaco, Massimo Monti, Alessandro Laviano, Maria Grazia Chiappini, Maurizio Muscaritoli

**Affiliations:** ^1^Department of Translational and Precision Medicine, Sapienza University of Rome, Rome, Italy; ^2^Department of Surgical Sciences, Sapienza University of Rome, Rome, Italy; ^3^Hemodialysis Unit, Fatebenefratelli Hospital, Rome, Italy; ^4^Advanced Molecular Diagnostic Unit, Istituto Dermopatico dell’Immacolata-IRCCS, Rome, Italy; ^5^Department of Public Health and Infectious Diseases, Sapienza University of Rome, Rome, Italy; ^6^Analytical Laboratory Unit, Sant’Andrea Hospital, Department NESMOS, Sapienza University of Rome, Rome, Italy

**Keywords:** hemodialysis, physical activity, body composition, beta-aminoisobutyric acid, longitudinal study

## Abstract

**Rationale:** Low physical activity is frequent in end stage renal disease. We evaluated the longitudinal change in physical activity and its barriers in hemodialysis (HD) patients and the association between the patterns of physical activity change, body composition, and beta-aminoisobutyric acid (BAIBA), as circulating myokine.

**Methods:** This is an observational study, where HD patients were considered in a 24-month follow-up. We assessed overtime the change of physical inactivity and its barriers by validated questionnaires, body composition by bioimpedance analysis, muscle strength by hand-dynamometer, and plasma BAIBA levels by liquid chromatography spectrometry. Parametric and non-parametric analyses were performed, as appropriate.

**Results:** Out of the 49 patients studied at baseline, 39 completed the first-year follow-up, and 29 completed the second year. At month 12, active patients had higher intracellular water (ICW) (*P* = 0.001) and cellular mass (*P* < 0.001), as well as at month 24 (*P* = 0.012, *P* = 0.002; respectively) with respect to inactive. A significant reduction in ICW was shown at month 12 (*P* = 0.011) and month 24 (*P* = 0.014) in all patients. The barrier “reduced walking ability” was more frequent in inactive patients with respect to active at month 12 (*P* = 0.003) and at month 24 (*P* = 0.05). At month 24, plasma BAIBA levels were higher among active patients with respect to inactive (*P* = 0.043) and a correlation was seen between muscle strength and ICW (*r* = 0.51, *P* = 0.005); normalizing BAIBA per body mass index, we found it lower with respect to baseline (*P* = 0.004), as well as after correcting per ICW (*P* = 0.001), as marker of muscle mass.

**Conclusion:** A high prevalence of physical inactivity persisted during a 24-month follow-up in this cohort. We found an association between physical activity and a decline in marker of muscularity and reduced plasma BAIBA levels.

## Introduction

The assessment of physical activity among hemodialysis (HD) patients is a crucial clinical aspect which has improved the understanding of the association between frailty and poor outcomes (Sy and Johansen, 2017). This assessment allows to recognize that inactive patients with end-stage renal disease (ESRD) undergoing maintenance HD are at higher risk for morbidity and mortality (Sy and Johansen, 2017; Ku et al., 2018). Physical inactivity is associated with the risk of falls, hospitalizations, cognitive impairment, vascular access failure, and poor quality of life among HD patients (Johansen et al., 2013a; Sy and Johansen, 2017) and exercise may be effective to reduce this risk (Johansen et al., 2013b). We recently investigated the presence of physical inactivity and its barriers among a cohort of HD patients and the association with the myokine beta-aminoisobutyric acid (BAIBA) (Molfino et al., 2017), which is formed by the catabolism of valine, determining several beneficial effects on muscle metabolism in an autocrine/paracrine manner (Molfino et al., 2017; Huh, 2018). In particular, BAIBA secretion increases with exercise and induces browning of white fat and hepatic beta-oxidation having potential implication in the physiological mechanisms of physical activity (Roberts et al., 2014).

Although low physical activity accompanies ESRD progression (Robinson-Cohen et al., 2014), few studies have documented the time course and the features of physical activity changes that occur with ESRD progression (Johansen et al., 2000).

The patterns of change in physical activity and barriers appear clinically relevant because they may provide insights into an appropriate timing of diagnosis and intervention to achieve the greatest impact on outcomes among HD patients. In addition, to date few data are available on the role of exercise-induced myokines, as possible biomarkers of physical activity and its variation in humans and in particular among HD patients who are known to be extremely sedentary (Johansen et al., 2000). In this light, research on possible novel biomarker(s), such as BAIBA, to easily assess and monitor muscularity associated or not to low physical activity, appears clinically important.

In this study, we first aimed to determine the longitudinal changes (up to 24 months) in physical activity levels among patients on HD, including barriers to physical activity. We next analyzed the association between these patterns of physical activity changes, BAIBA levels, body composition, and muscle strength.

## Patients and Methods

This longitudinal study was performed on patients from the Dialysis Unit of “Fatebenefratelli” Hospital, Isola Tiberina, in Rome, Italy. Women and men ≥18 years chronically maintained on HD, participating on a study investigating the role of nutrition and quality of life, whose baseline characteristics were previously described (Molfino et al., 2017), underwent to a follow-up of 24 months. In summary, 52 patients are chronically treated at the HD center and the inclusion criteria consisted in enrolling the entire HD cohort with the exception of patients with highly catabolic diseases, such as cancer, chronic infections, and AIDS, and the absence of informed consent, as previously shown (Molfino et al., 2017). The sample size we used was representative of the entire number of patients, respecting the inclusion criteria, treated at the single Dialysis Unit.

The study was approved by the local ethics committee. Written informed consent was obtained from all patients. Patients were maintained on a regular HD prescription, three times a week, for 4-h sessions during the 2-year follow-up.

### Patient Clinical Characteristics

Participants’ demographic and clinical characteristics, including age, gender, weight, height, body mass index (BMI), dialysis vintage, and Kt/V were recorded, and comorbidities and current therapies were collected. Nutritional and inflammatory biomarkers were assessed at baseline (Molfino et al., 2017), at 12 and 24 months.

### Body Composition Analysis

Body impedance analysis (BIA) was performed in all HD patients to assess body composition overtime (Model 101, single-frequency BIA, Akern, Florence, Italy). Blinded research staff performed BIA 1 h after the end of the second HD session of the week. As previously shown, the patient maintained a supine position during this period (Jankowska et al., 2006; Molfino et al., 2017). The pairs of electrodes were placed on the non-access side of the body on the hand to the foot for injecting current and on the wrist to the ankle for measuring voltage. Total body water was estimated using the resistance extrapolated to frequency and parameters, including intracellular water (ICW) and cellular mass, as the metabolically active component of lean tissue mass, were calculated using a program provided by the producer (Jankowska et al., 2006; Molfino et al., 2017).

### Physical Activity Level

As for baseline (Molfino et al., 2017), at month 12 and month 24, we asked enrolled patients to answer questions on the self-reported frequency of physical activity, including recreational walking, running, and so on, during leisure time. This questionnaire was shown to be reliable in large cohorts of dialysis patients (Tentori et al., 2010; Delgado and Johansen, 2012).

A second questionnaire was used to evaluate the barriers to physical activity in general or to lower physical activity levels than desired, investigating 23 barriers, designed on the basis of previously validated questionnaire in the HD population (Delgado and Johansen, 2012; Johansen et al., 2014b).

Patients who experienced exercise at least once a week were considered active, those who never exercised or reported physical exercise less than once a week were considered inactive (Delgado and Johansen, 2012; Johansen et al., 2014b).

### Muscle Strength

The muscle function was assessed on the same day of the recruitment immediately before starting the HD session by handgrip strength measurement, using a handle dynamometer (JAMAR, Sammons Preston Rolyan, Bolingbrook, IL, United States). The test was performed conducting three attempts with each hand, and the mean of the strongest hand was used to determine muscle strength (Johansen et al., 2014a; Molfino et al., 2017).

### Plasma BAIBA Levels Assay

Blood samples were obtained from the HD patients immediately before the second HD session of the week and kept at room temperature for 1 h before the plasma was isolated. Aliquots of plasma were stored at −80°C until analysis. Taking into account the expected association between the myokine BAIBA and body size and body composition, in particular muscularity, we also used BAIBA/BMI and more specifically BAIBA/ICW (%) ratio (ICW, as marker of muscularity) (Johansen et al., 2014a) in our analyses. Data presented here utilize the mean of two measurements.

Plasma BAIBA levels were assessed by a Liquid chromatography–tandem mass spectrometry (LC–MS/MS) method, as previously described (Molfino et al., 2017). Briefly, 40 μL of plasma samples were added to 160 μL of internal standard in acetonitrile. The samples were vortex-mixed, centrifuged and 100 μL of clean upper layer were then diluted with 100 μL of 0.1% formic acid and transferred in autosampler vials. Chromatographic separation was achieved using a Phenomenex Luna HILIC column (100 × 2.0 mm, 3 μm; Phenomenex, Torrance, CA, United States), with an elution gradient (Molfino et al., 2017). The HPLC-MS/MS analysis were performed using an Agilent Liquid Chromatography System series 1100 (Agilent Technologies, Santa Clara, CA, United States) coupled with a 3200 triple quadrupole system (Applied Biosystems, Foster City, CA, United States), with a Turbo Ion Spray source. Data were acquired and processed by Analyst 1.5.1 Software.

### Statistical Analyses

Patient characteristics were described using mean (±SD) for continuous normally distributed variables and percent for dichotomous variables. Variables that were not normally distributed were described using median with 25th and 75th percentiles. The proportion of patients self-reporting each barrier and the average number of barriers perceived per patient were calculated. Chi-square, Mann–Whitney, and Kruskall–Wallis tests were used to study the association between reported barriers to physical activity and patient characteristics, the association between barriers and reported levels of physical activity, between barriers and time (baseline, 12, 24 months) and between BAIBA, levels of physical activity and its barriers, body composition and muscle strength overtime. Spearman’s correlation index was used to assess associations between continuous measurements. A linear mixed-effect model was used to evaluate the change of body composition overtime, as well as a model to evaluate changes of clinical parameters and biomarkers overtime. The model included physical activity, categorical time point, physical activity level per time point interaction as fixed effect and subject as a random effect.

A standard two-tailed a (*P* < 0.05) was considered statistically significant. Statistical analysis was performed in R v.3.4.3.

## Results

### Patient Characteristics

Characteristics of the participants are reported in Table 1.

**TABLE 1:** Patient characteristics.

	**Month 12**	**Month 24**	
	
	**Patients *N* = 39**	**Patients *N* = 29**	**P**
Sex (male)	31	23	
Age, years	66 ± 15.2	66.9 ± 14.7	0.85
BMI, weight (kg)/height ${}^{2}$ (m)	24.5 ± 5.3	25.0 ± 5.82	0.87
Hb, g/dl	11.2 ± 1.3	11.4 ± 1.22	0.96
Albumin, g/l	39.7 ± 3.04	37.4 ± 4.08	0.49
Urea^#^, mg/dl	118.6 ± 31.8	132.6 ± 36.1	0.68
Creatinine, mg/dl	9.1 ± 2.5	8.9 ± 2.4	0.41
CRP, mg/dl	0.4 (0.2; 1.3)	0.37 (0.19; 0.9)	0.97
Dialysis vintage, months	40.5 (25; 70.2)	50 (37; 77)	0.21
nPCR, g/kg/d	0.92 ± 0.2	0.94 ± 0.17	0.97
Kt/V	1.29 ± 0.24	1.34 ± 0.26	0.39
Physical inactivity (yes)	20 (51%)	19 (63%)	0.27

*^#^Urea levels are 2.14 times the corresponding BUN levels. BMI, body mass index; BUN, blood urea nitrogen; CRP, C-reactive protein; Hb, hemoglobin; nPCR, normalized protein catabolic rate.*

In summary, 49 patients (13 women) were studied at baseline with a mean age of 66.6 ± 15.5 years. Median dialysis vintage was 37 months [interquartile range (IQR), 13–92] with a mean Kt/V of 1.3 ± 0.3 (Molfino et al., 2017) and 20% of patients were on hemodiafiltration.

At 12-month follow-up, 7 patients died, 2 were transferred to another dialysis facility and 1 patient received renal transplantation. Therefore, 39 patients were considered, with a mean age of 66 ± 15.1 years and a median dialysis vintage of 40.5 months (IQR, 25–70.2) (Table 1).

At 24-month follow-up, 5 patients died, 2 were transferred to another dialysis facility and 3 patients received renal transplantation. A total of 29 patients were studied, with a mean age of 66.9 ± 14.7 years and a median dialysis vintage of 50 months (IQR, 37–77) (Table 1).

During the entire follow-up period, as for baseline (Molfino et al., 2017), more than 50% of the patients were treated with antihypertensive therapies, statins, vitamin supplements, erythropoietin, and potassium, and phosphate binders and supplemented with folic acid.

### Physical Activity Level and Its Change

At baseline, inactive patients were 51 and 88% reported having barriers to physical activity (Molfino et al., 2017). Similarly, at 12 months 51% of the patients reported performing physical activity during leisure time less than once per week, almost never, or never, and therefore were considered inactive, and 90% reported barriers to physical activity. Four patients, who were active at baseline, resulted inactive at month 12 months.

At month 24, 65% of patients resulted inactive and 96% reported having barriers to physical activity. From baseline to month 24, according to the questionnaire, 7 active patients resulted inactive. In the entire cohort of the enrolled patients, the median number of barriers endorsed at baseline (*n* = 4), month 12 (*n* = 3), and month 24 (*n* = 3) did not significantly change overtime (*P* = 0.30). From baseline to 24-month follow-up, the type of barriers did not significantly change in all HD patients and also when considering active and inactive patients separately. We did not observe differences between active and inactive patients in terms of type and numbers of comorbidities, and in nutritional and inflammatory biomarkers. In addition, we did not find a significant association between inactivity at baseline and mortality (*P* = 0.114).

During the entire follow-up, the most frequently endorsed barrier was “fatigue on dialysis days.” The barrier “reduced walking ability” was confirmed to be more frequent in inactive patients with respect to active patients at month 12 (*P* = 0.003) and month 24 (*P* = 0.05).

### Body Composition and Physical Activity Change

Differences in body composition parameters between active and inactive patients during the follow-up are reported in Table 2. As for baseline (Molfino et al., 2017), at month 12, we found that active patients had higher ICW (%) (*P* = 0.001) and cellular mass (%) (*P* < 0.001) with respect to inactive patients. Similarly, at month 24 active patients showed compared to inactive patients higher ICW (%) (*P* = 0.012) and cellular mass (%) (*P* = 0.002) (Table 2).

**TABLE 2:** Biompendance analysis parameters.

	**Baseline**			**12-month follow-up**			**24-month follow-up**		
	**Active patients**	**Inactive patients**	**P**	**Active patient**	**Inactive patients**	**P**	**Active patient**	**Inactive patients**	**P**
	***N* = 24 (49%)**	***N* = 25 (51%)**		***N* = 19 (49%)**	***N* = 20 (51%)**		***N* = 10 (35%)**	***N* = 19 (65%)**	
Body weight (Kg)	69.9 ± 18.4	69.2 ± 14.7	0.88	66.6 ± 18.0	71.2 ± 13.7	0.38	62.25 ± 13.8	73.65 ± 16.9	0.07
*Biompendance analysis*									
Fat mass									
(%)	33.2 ± 8.9	31.6 ± 8.0	0.52	30.3 ± 8.2	30.5 ± 6.9	0.94	32.4 ± 9.1	32.7 ± 7.4	0.98
(kg)	23.3 ± 9.2	21.0 ± 7.2	0.58	20.35 ± 8.07	20.83 ± 7.87	0.85	20.37 ± 7.54	24.2 ± 8.12	0.21
Intracellular water (%)	51.2 ± 7.3	44.5 ± 9.5	0.008	52.4 ± 5.98	43.1 ± 9.4	0.001	53.1 ± 5.97	45.4 ± 7.7	0.012
Cellular mass (%)	42.3 ± 7.3	35.9 ± 7.8	0.005	43.1 ± 4.02	33.04 ± 9.4	<0.001	43.08 ± 4.2	35.9 ± 6.3	0.002
Body mass cell index (kg/m^2^)	6.7 ± 1.9	5.6 ± 1.8	0.04	6.88 ± 1.9	5.65 ± 1.9	0.059	6.18 ± 1.72	6.95 ± 4.2	0.58
Phase angle (°)	5.2 ± 1.2	4.1 ± 1.1	<0.001	5.2 ± 1.0	4.0 ± 1.1	<0.001	5.4 ± 1.1	4.3 ± 1.2	0.02

Independent of the presence of physical inactivity, our cohort of HD patients showed, compared to baseline, a significant reduction in ICW (%) at month 12 (*P* = 0.011) and at month 24 (*P* = 0.014).

This trend overtime was confirmed after adjusting for the presence of physical inactivity, although at the limit of significance (at month 12, *P* = 0.046 and at month 24, *P* = 0.059, respectively).

### BAIBA, Physical Activity Level and Its Barriers, Body Composition and Muscle Strength Overtime

**FIGURE 1:**
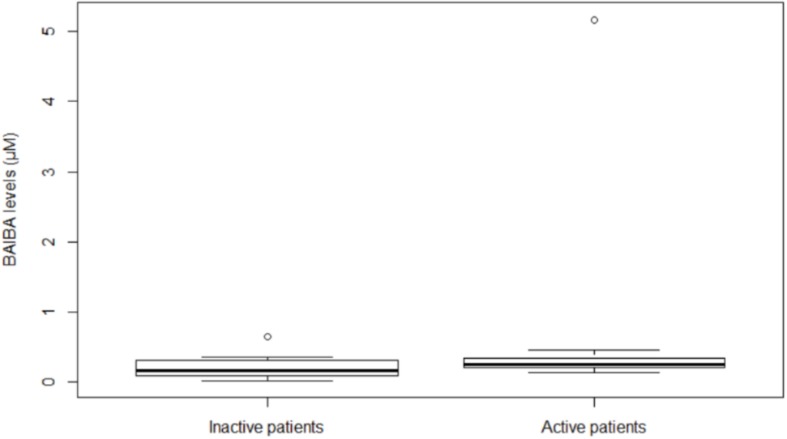
Plasma BAIBA levels in hemodialysis inactive and active patients at month 24. Box plot of BAIBA levels (μM) stratified by inactive or active patients. BAIBA levels were significantly higher in active with respect to inactive patients (*P* = 0.043). Lines represent the median, 25th and 75th percentiles, and the whiskers (error bars) below and above the box indicate the 10th and 90th percentiles. BAIBA, beta-aminoisobutyric acid.

In all the participants, at baseline BAIBA (μM) median value was 0.40 (IQR, 0.21–0.61) (Molfino et al., 2017) and at month 24 was 0.21 (IQR, 0.10–0.32). During the 24-month follow-up, BAIBA median level did not significantly change among all the participants; however, at month 24, median BAIBA level was higher among active patients with respect to inactive (Figure 1) (*P* = 0.043).

No significant association was found between plasma BAIBA levels and the number and type of barriers at month 24.

**FIGURE 2:**
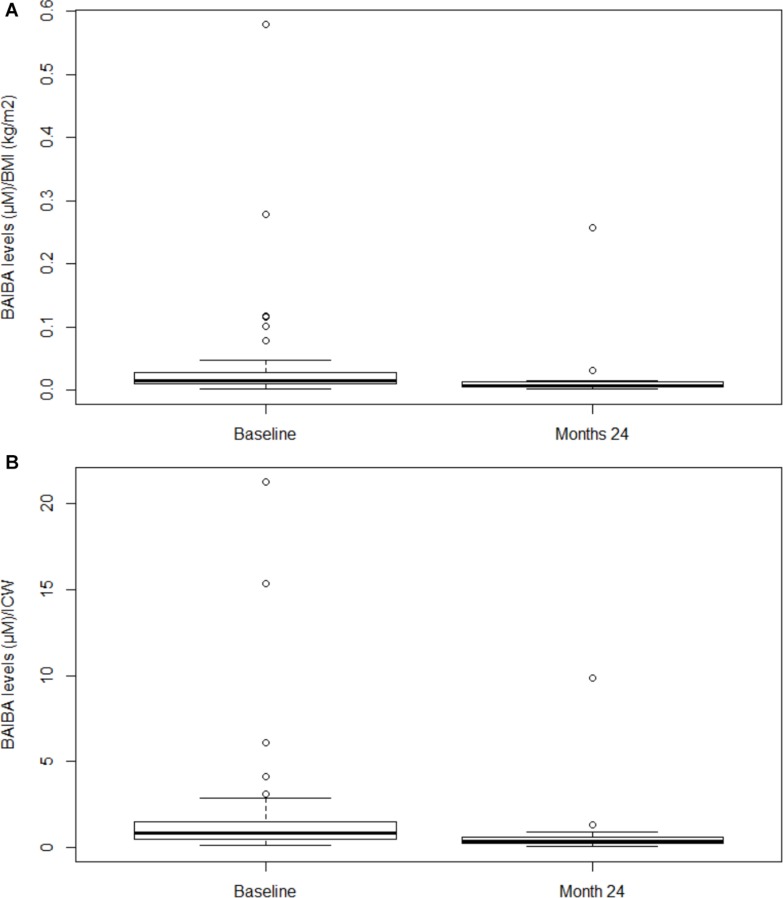
Plasma BAIBA levels normalized per BMI and per ICW in hemodialysis patients at baseline and at month 24. In panel **(A)** Box plot of BAIBA/BMI ratio at baseline vs. month 24 (*P* = 0.004). Lines represent the median, 25th and 75th percentiles, and the whiskers (error bars) below and above the box indicate the 10th and 90th percentiles. BAIBA, betaaminoisobutyric acid; BMI, body mass index. In panel **(B)** Box plot of BAIBA/ICW ratio at baseline vs. month 24 (*P* = 0.001). Lines represent the median, 25th and 75th percentiles, and the whiskers (error bars) below and above the box indicate the 10th and 90th percentiles. BAIBA, betaaminoisobutyric acid; ICW, intracellular water.

Independent of the presence of physical inactivity, when considering BAIBA normalized per BMI (BAIBA/BMI), we found this ratio at month 24 significantly lower with respect to baseline (*P* = 0.004) (Figure 2A). Similarly, when BAIBA was normalized per ICW (BAIBA/ICW), we documented this ratio lower with respect to baseline (*P* = 0.001) (Figure 2B).

At month 24, median values of hand-grip measurement were not different between active (21 mmHg) and inactive patients (21 mmHg). Moreover, no association was documented overtime between muscle strength and BAIBA levels, physical inactivity and its barriers. However, at month 24, a significant correlation was observed between muscle strength and ICW (*r* = 0.51, *P* = 0.005).

## Discussion

There have been robust data on physical inactivity and its association with frailty during HD and how this condition may affect patient’s prognosis (Johansen et al., 2013b, 2016; Sy and Johansen, 2017). This suggests to physicians to evaluate physical activity level to prevent clinical deterioration and functional dependence during ESRD (Rodríguez-Mañas et al., 2013). The prevalence of HD patients physically inactive and sedentary is high (Johansen et al., 2007, 2014b), and its percentage depends on how physical activity is defined (Delgado and Johansen, 2012; Bossola et al., 2014; Johansen et al., 2016).

We previously confirmed in a cohort of HD patients a high prevalence of physical inactivity, defined using self-reported physical function (Molfino et al., 2017). The majority of the data available in the literature on this topic have been obtained in cross-sectional studies (Johansen et al., 2014a, 2016). Novel information may be obtained by longitudinal studies by comparing the timing of changes in body composition with the development of several conditions, including low muscle mass and frailty (Johansen et al., 2014a). In this light, longitudinal data are important to ascertain whether modifications in body composition are strictly related with physical activity changes and whether possible biomarker(s) of muscularity may identify or predict changes in body composition and in physical activity level which are associated with adverse outcomes.

In the present study, we evaluated overtime (up to 24 months) in a single-center cohort of HD patients, being representative of a homogenous population, the physical activity level during leisure time. We administered, as for baseline (Molfino et al., 2017), a first questionnaire on the self-reported frequency of physical activity and a second questionnaire regarding barriers to physical activity in general or to lower physical activity levels than desired. The participation in physical activity, already low at baseline (Molfino et al., 2017), was confirmed to be reduced after the 24-month follow-up, as 63% of the patients reported participating in light activity never, almost never, or less than once per week, and 96% of patients reported endorsing barriers to physical activity. Interestingly, although not statistically different, the median number of barriers at month 24 was lower than baseline. Considering that low level of physical activity is associated with reduced survival, we believe that those patients in our cohort who survived at month 24 may be less inactive. The most frequent barriers observed at month 12 and 24 were “fatigue on dialysis day” and “reduced walking ability.” The barrier “reduced walking ability” was more frequently reported by inactive patients with respect to active during the entire follow-up at month 12 and 24. This highlights the importance of walking difficulty in conditioning reduced mobility in HD patients. Moreover, results of a recent multicenter study documented that barriers to physical activity and non-proactive health-care staff’s attitude reduced the implementation of physical activity in the HD population and that patients not reporting barriers were those who showed greater beneficial effects from a proactive staff’s attitude (Regolisti et al., 2018).

Physical inactivity may be the cause and/or the effect of body composition derangements. In fact, low activity level may determine and/or worsen low muscle mass; on the other hand, lower muscularity may reduce physical performance. In this light, it appears advisable to evaluate body composition overtime in association with the level of physical activity.

During our follow-up, we found that lower ICW, a proxy of muscle mass (Johansen et al., 2014a), was associated with physical inactivity at month 12 and 24. A significant decline in ICW was observed from baseline to month 24 in the entire cohort. This is in line with the results obtained in a larger cohort of HD patients where lower ICW was associated with frailty (Johansen et al., 2014a).

When performing a multivariable analysis accounting for physical activity, the trend of ICW reduction overtime was confirmed in the inactive group, suggesting the possibility that the time-altered body composition association might be explained, at least in part, by physical inactivity.

In addition, we focused on the possible relationship between changes overtime of body composition, physical activity, and BAIBA, as circulating biomarker of muscularity during HD. This aspect appears relevant considering that BAIBA seems to present different actions within skeletal muscle, including attenuation of insulin resistance, inflammation and inducing fatty acid oxidation (Jung et al., 2015). Not conclusive data are yet available on the physiology/pathophysiology of BAIBA and we do not have information on BAIBA removal from HD. However, Roberts et al. showed robust data in humans documenting that BAIBA levels increased with physical activity and were inversely related with metabolic risk factors (Roberts et al., 2014).

In the present study, at the end of the follow-up (month 24), inactive patients showed lower plasma BAIBA levels with respect to active patients and, independent of the level of physical activity, we found lower BAIBA/BMI ratio with respect to baseline. This trend was confirmed when BAIBA was corrected per marker of muscle mass. Although not at baseline, a correlation was present at month 24 between muscle mass and its function (muscle strength). However, as for baseline, no correlation was seen between BAIBA and muscle function indicating a possible different mechanism(s) regulating BAIBA expression and muscle mass and its function. In particular, our results did not allow us to ascertain a cause-effect relationship between plasma BAIBA levels and changes of physical activity and muscularity overtime. However, our data give us the perspective to possibly use BAIBA as a muscle-specific biomarker that might help physicians in monitoring muscularity in the HD population.

Our study has several limitations, including the patient sampling (single center study) and, in particular, the reduced sample size at month 12 and 24, possibly affecting the investigated associations, and the study group is highly variable including a wide range of dialysis vintage. As for baseline, physical activity is only subjectively assessed by the patients and not by more objective instruments such as accelerometers or pedometers, and the tool used to evaluate physical activity did not include questions on potential additional activities (Molfino et al., 2017). The association found between physical inactivity and body composition does not imply a clear causality in one or other direction, as previously suggested by others (Johansen et al., 2014a). We could not determine if low ICW caused physical inactivity or if being more sedentary determined body composition derangements. Using BIA, possible errors in estimating body composition and fluid distribution remain (Kyle et al., 2004). Moreover, the biomarker BAIBA has not yet been validated in HD population, with poor availability of information regarding its accumulation in HD patients and its removal with dialysis.

## Conclusion

In conclusion, we observed in our cohort of HD patients a high prevalence of physical inactivity during a 24-month follow-up and a clinically relevant number of endorsed barriers. These conditions were associated with a decline in markers of muscularity overtime. The levels of the myokine BAIBA after 24-month follow-up were significantly lower among inactive patients and, independent of the level of physical activity, BAIBA levels significantly reduced overtime when corrected per index of muscularity. Larger clinical investigations are essential to clarify the role of BAIBA and body composition changes overtime in HD, as well as to assess BAIBA levels evaluating its changes after interventional studies by administering nutritional support and/or physical activity interventions.

## Ethics Statement

Ethics Committee of Fatebenefratelli Hospital, Rome, Italy, approved the protocol. Written informed consent was obtained from each participant. All subjects gave written informed consent in accordance with the Declaration of Helsinki.

## Author Contributions

AM designed the research, conducted the research, analyzed the data, and wrote the manuscript. MIA conducted the research, collected the data, and wrote the manuscript. TA and AG collected the data. AS analyzed the data and performed the statistical analyses. LL performed the laboratory dosage. MS analyzed the laboratory data. SL conducted the research. MMo reviewed the manuscript. AL designed the research and reviewed the manuscript. MGC conducted the research, enrolled the patients, and reviewed the manuscript. MMu designed the research, reviewed the manuscript, and had primary responsibility for the final content.

## Conflict of Interest Statement

The authors declare that the research was conducted in the absence of any commercial or financial relationships that could be construed as a potential conflict of interest. The handling Editor declared a shared affiliation, though no other collaboration, with several of the authors, AM, MIA, SL, AS, MS, MMo, AL, and MMu, at the time of review.
